# The epidemic of acute lymphoid leukemia in China: current trends and future prediction

**DOI:** 10.3389/fonc.2023.1195065

**Published:** 2023-06-16

**Authors:** Wenxuan Zhu, Shixuan Liu, Ying Shi, Qingyu Tang, Jianzhong Sun, Ruhai Bai, Zhonghe Sun, Zhaoqing Du

**Affiliations:** ^1^ Department of Hepatobiliary Surgery, Shaanxi Provincial People’s Hospital, Xi’an, Shaanxi, China; ^2^ Health Science Center, Xi’an Jiaotong University, Xi’an, Shaanxi, China; ^3^ Department of Chronic Disease, Xi'an Weiyang District Center for Disease Control and Prevention, Xi’an, Shaanxi, China; ^4^ School of Public Affairs, Nanjing University of Science and Technology, Nanjing, Jiangsu, China; ^5^ Nanjing First Hospital, Nanjing Medical University, Nanjing, China

**Keywords:** acute lymphoid leukemia, leukemia, incidence, mortality, age-period-cohort effect, projection

## Abstract

**Background:**

China has experienced one of the fastest increases in the incidence of acute lymphoid leukemia (ALL). The aim of this study was to assess the long-term trends of the incidence and mortality of ALL in mainland China between 1990 and 2019 and to project these trends through 2028.

**Methods:**

Data on ALL were extracted from the Global Burden of Disease Study 2019; population data were extracted from World Population Prospects 2019. An age–period–cohort framework was used in the analysis.

**Results:**

The net drift for the incidence of ALL was 7.5% (95% confidence interval [CI]: 7.1%, 7.8%) per year in women and 7.1% (95% CI: 6.7%, 7.6%) in men, and local drift was found to be higher than 0 in every studied age group (p<0.05). The net drift for mortality was 1.2% (95% CI: 1.0%, 1.5%) in women and 2.0% (95% CI: 1.7%, 2.3%) in men. Local drift was lower than 0 in boys aged 0–4 years and girls aged 0–9 years and higher than 0 in men aged 10–84 years and women aged 15–84 years. The estimated period relative risks (RRs) for both incidence and mortality showed increasing trends in the recent period. The cohort RRs for incidence showed increasing trends in both sexes; however, the cohort RR for mortality was decreased in the recent birth cohort (women born after 1988–1992 and men born after 2003–2007). Compared with that in 2019, the incidence of ALL in 2028 is projected to increase by 64.1% in men and 75.0% in women, and the mortality is predicted to decrease by 11.1% in men and 14.3% in women. The proportion of older adult/adults individuals with incident ALL and ALL-related death was projected to increase.

**Conclusions:**

Over the last three decades, the incidence and mortality rates of ALL have generally increased. It is projected that the incidence rate of ALL in mainland China will continue to increase in the future, but the associated mortality rate will decline. The proportion of older adult/adults individuals with incident ALL and ALL-related death was projected to increase gradually among both sexes. More efforts are needed, especially for older adult/adults individuals.

## 1 Introduction

Acute lymphoid leukemia (ALL) is a common blood cancer (1) caused by abnormal monoclonal proliferation and expansion of immature lymphoid cells in the bone marrow, blood, and other organs (2). These cells accumulate within the bone marrow, leading to suppression of growth and differentiation of normal blood cells (3). ALL usually progresses rapidly, with symptoms including anemia, fever, bleeding, and swelling of the lymph nodes (4), and ALL can lead to death quickly if left untreated (5). ALL is a very serious disease; once leukemia cells enter the peripheral bloodstream, almost any organ system can be affected (2). The treatment methods are complex, and the treatment effect is limited. Current treatment strategies for ALL include chemotherapy and intensive multimodal treatment regimens such as high-dose chemotherapy, stem cell rescue, and radiation therapy (6). Long-term survival at 10 years in children is 63% and in adults, 25–35% (7). As the 10th most common diagnosed cancer, there were 518,485 new cases and 347,583 deaths of ALL globally in 2017 (8). In the past three decades, the age-standardized incidence of ALL decreased slightly from 0.89 per 100,000 population in 1990 to 0.85 per 100,000 population in 2019 globally (9), and the age-standardized mortality rate increased from 37.26 per 100,000 population to 55.22 per 100,000 population (9).

Unlike the global trend, China has experienced a rapid increase in the incidence of ALL in the past few decades (9). According to the Global Burden of Disease study (GBD) 2019, incident cases of ALL in mainland China accounted for approximately 37% of global incident cases and approximately 25% of global deaths (10). However, few studies have evaluated the long-term burden of ALL in China, and the potential effects of these trends are still unknown (e.g., age, period, and cohort effect). To address these limitations, we used the age–period–cohort (APC) framework to assess long-term trends of ALL in mainland China. To better understand the burden of ALL in the future, we also projected future incidence and mortality rates of ALL. Furthermore, we decomposed the age composition of the future ALL rate. The results of this study provide useful information to help understand the burden of ALL in mainland China and may provide useful guidance for further prevention and control of ALL.

## 2 Method

### 2.1 Data sources

The data used in this study were extracted from the GBD 2019, a large international cooperative project that globally, regionally, and nationally assessed age- and sex-specific mortality rates for 369 diseases and injuries in 204 countries and territories from 1990 to 2019 (10). There were five main data sources that GBD 2019 utilized to summarize data on causes of death in China: the Chinese Center for Disease Control and Prevention, the National Office for Maternal and Child Health Surveillance, the Center for Health Statistics and Information at the National Health Commission, the National Office for Cancer Control and Prevention, and individual researchers at academic institutions worldwide (11). National surveys, cancer registries, hospital inpatient data, published studies, and the Chinese Center for Disease Control and Prevention cause of death reporting system were used to estimate the incidence of non-fatal outcomes in China, and the Bayesian meta-regression method was used in the estimation process (11). ALL was defined in this study according to the 9th and 10th revisions of the International Classification of Diseases (ICD10 codes: C91-C91.0, C91.2-C91.3, C91.6, C92-C92.6, C93-C93.1, C93.3, C93.8, and C94-C95.9; ICD9 codes: 204-204.0, 204.2, 205-205.3, 206-206.1, and 207-208.9) (10). To characterize the temporal trends in China, the GBD 2019 global age-standard population was used to calculated age-standardized rates.

The population data extracted from the GBD 2019 were used in the trend analysis. Predicted population data extracted from United Nations World Population Prospects 2019 were used to make future predictions of incidence and mortality.

### 2.2 Statistical analysis

To investigate the trends in the incidence and mortality rates of ALL, an APC model was constructed. This statistical model is generally used in epidemiological studies and distinguishes among the effects of age, period, and cohort, which are time-related variables. The specific equation is as follows (12):


$$Y=log\left(M\right)=b+mAge_{i}+nPeriod_{j}+pCohort_{k}+e$$

where Y denotes the outcome; *m*, *n*, and *p* denote the coefficients of age, period, and cohort in the APC model, respectively; *b* denotes the intercept of the model; and *e* denotes the residual of the APC model.

By using the APC framework, the following functions were estimated: net drift represents the annual percentage change in ALL incidence or mortality; local drift represents the annual percentage change in the incidence or mortality in each age group; and the longitudinal age curve reflects the age effect and represents expected age-specific incidence or mortality in the reference birth cohort adjusted for period effects. Period relative risk (RR) represents the period effect and reflects the incidence or mortality risk for certain time periods. Cohort RR represents the cohort effect and reflects the incidence or mortality risk in different birth cohorts (13).

To conduct the trends analysis, incidence and mortality data were grouped into consecutive 5-year periods from 1990–1994 (median, 1992) to 2015–2019 (median, 2017). A total of 18 successive 5-year age intervals from 0 to 4 years to 85 to 89 years were included. A total of 23 consecutive cohorts were classified, including those born from 1903–1907 (median, 1905) to 2013–2017 (median, 2015). In this study, the median age group, period, and birth cohort were set as references. Estimable parameters were estimated by the American National Cancer Institute APC web tool (Biostatistics Branch, National Cancer Institute, Bethesda, MD, USA) (14). Wald chi-square tests were used to determine the significance of the estimable functions.

To predict the incidence and mortality in the future, the Bayesian APC method was used. The Bayesian APC model provides higher coverage and has a better performance in making projections (15). By developing a Bayesian APC framework, integrated nested Laplace approximation approach was used (16); this method can approximate Bayesian inference and is a fast alternative to the Markov chain Monte Carlo method for fitting Bayesian models. We used the R package “BAPC” to fit the Bayesian APC model, and a random walk of second order was used to model age, period, and cohort effects. To fit the Bayesian APC model, those with incident cases and associated deaths were grouped into 18 consecutive 5-year age intervals from 0 to 4 years to 85 to 89 years in every year from 1990 to 2019; population data were also grouped into 18 consecutive 5-year age groups from 1990 to 2028. All analyses were performed in R 4.1.2. A p-value<0.05 was statistically significant.

## 3 Results

### 3.1 Annual percentage changes in acute lymphoid leukemia incidence and mortality

**Figure 1:**
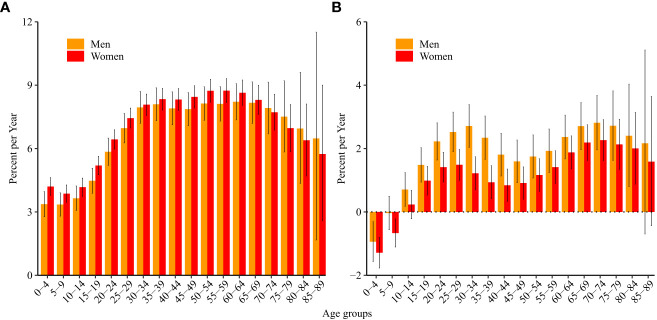
Local drift values for acute lymphoid leukemia **(A)** incidence and **(B)** mortality in China. Local drift, age-specific annual percentage changes in the acute lymphoid leukemia incidence/mortality rates and corresponding 95% confidence intervals.

Net drift indicates the annual percentage change in the expected age-adjusted rate over time. Local drift indicates the annual percentage change in the expected rate in each age group over time. The net drift for ALL incidence was 7.5% (95% CI: 7.1%, 7.8%) per year in women and 7.1% (95% CI: 6.7%, 7.6%) in men. All local drift values were higher than 0 (significantly with p<0.05), which means that the incidence of ALL increased in every age group from 0 to 89 years during the last 30 years (**Figure 1A**).

The net drift for ALL mortality was 1.2% (95% CI: 1.0%, 1.5%) in women and 2.0% (95% CI: 1.7%, 2.3%) in men. The local drift was less than 0 in boys aged 0–4 years and girls aged 0–9 years (significantly with p<0.05), which means that mortality due to ALL decreased over the last 30 years in these age groups. The local drift was higher than 0 in men aged 10–84 years and women aged 15–84 years (**Figure 1B**).

### 3.2 Longitudinal age curves of acute lymphoid leukemia incidence and mortality

**Figure 2:**
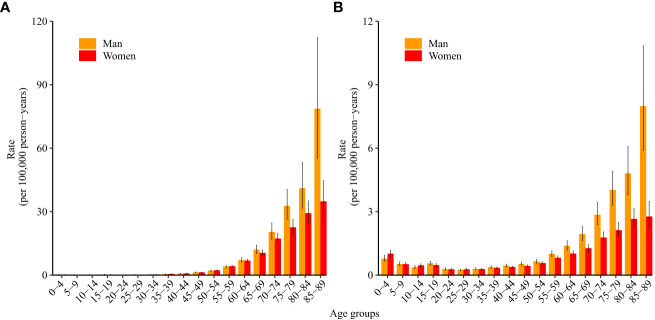
Longitudinal age curves of acute lymphoid leukemia **(A)** incidence and **(B)** mortality in China. Fitted longitudinal age-specific rates of acute lymphoid leukemia incidence/mortality (per 100,000 person-years) and corresponding 95% confidence intervals (some of them were too narrow to show in the figure).

**Figure 2** shows the longitudinal age curves of ALL incidence (**Figure 2A**) and mortality (**Figure 2B**). For incidence, in the same birth cohort, children aged 10–14 years had the lowest incidence of ALL, and the incidence increased with age and increased rapidly after the age of 60 years. After the age of 70, the incidence of ALL in men was higher than that in women (p<0.05).

For mortality, in the same birth cohort, women and men aged 25–29 years had the lowest mortality due to ALL, and the mortality increased with age and increased rapidly after the age of 55 years. After the age of 60, mortality due to ALL in men was higher than that in women (p<0.05).

### 3.3 Period and cohort relative risks of acute lymphoid leukemia incidence and mortality

**Figure 3:**
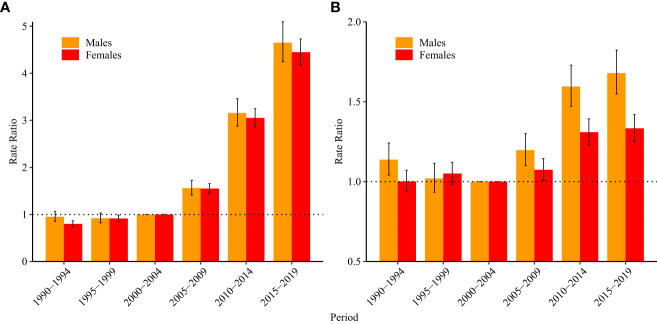
Period relative risks of acute lymphoid leukemia **(A)** incidence and **(B)** mortality rate by sex in China. The relative risks for each period compared with the reference period (years 2000–2004) adjusted for age and non-linear cohort effects and corresponding 95% confidence intervals.

As shown in **Table 1**, the period RRs were statistically significant for both sexes (p<0.05). **Figure 3A** shows the estimated period RRs for incident ALL in women and men. In women, the period RR showed a monotonic increasing trend, while the period RR in men showed a monotonic increasing trend only after 1995. From 1990–1994 to 2015–2019, the RRs increased by 455.8% and 388.7% in women and men, respectively. For mortality, the period RRs for both women and men showed upward trends after 2000. From 1990–1994 to 2015–2019, the RRs increased by 33.2% and 47.7% in women and men, respectively (**Figure 3B**).

**Table 1:** The Wald chi-square test for estimable functions in the age–period–cohort model.

	Null hypothesis	Male		Female	
		Chi-square	p-value	Chi-square	p-value
Incidence					
	All period RR^†^ = 1	203.7	<0.001	3,297.4	<0.001
	All cohort RR = 1	2004.4	<0.001	3,692.8	<0.001
	All local drifts^‡^ = net drift^§^	1501.5	<0.001	357.4	<0.001
Mortality					
	All period RR = 1	262.3	<0.001	152.5	<0.001
	All cohort RR = 1	203.0	<0.001	185.4	<0.001
	All local drifts = net drift	93.4	<0.001	124.1	<0.001

^†^RR, rate ratio.

^‡^Local drift, annual percentage change in the expected age-specific rates over time.

^§^Net drift, annual percentage change in the expected age-adjusted rates over time.

**Figure 4:**
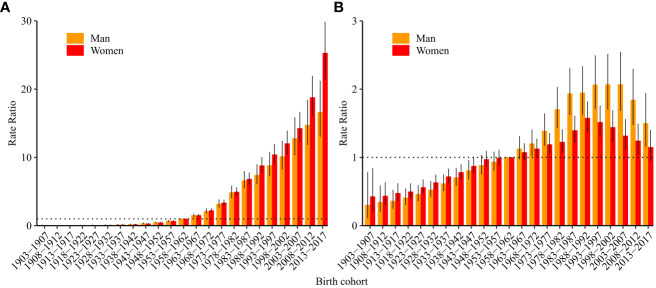
Cohort relative risks of acute lymphoid leukemia **(A)** incidence and **(B)** mortality rate by sex in China. The cohort relative risks for each birth cohort compared with the reference cohort (birth cohort, 1953–1957) adjusted for age and non-linear cohort effects and corresponding 95% confidence intervals.

According to the results of Wald tests, the cohort RRs were statistically significant for both sexes (p<0.05) (**Table 1**). **Figure 4A** shows the estimated cohort RRs for incident ALL in women and men. Cohort RRs for incident ALL in both men and women showed continuous increases. For mortality (**Figure 4B**), cohort RRs decreased in women born after 1988–1992 and in men born after 2003–2007.

### 3.4 Projected future incidence and mortality due to acute lymphoid leukemia

**Figure 5:**
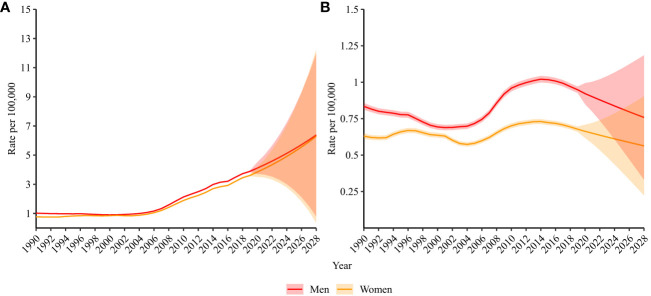
Projected **(A)** incidence and **(B)** mortality rate of acute lymphoid leukemia in China. The incidence and mortality rates were standardized to the GBD 2019 (Global Burden of Disease Study 2019) global age-standard population.

**Figure 5** shows predictions of the incidence (**Figure 5A**) and mortality (**Figure 5B**) of ALL in mainland China. The incidence rate of ALL was 3.9 (95% CI: 3.8, 3.9) per 100,000 population and 3.6 (95% CI: 3.6, 3.7) per 100,000 population in 2019 in men and women aged 0–89 years, respectively. It is projected that the incidence rate will increase to 6.4 (95% CI: 0.8, 12.0) per 100,000 population (increased by 64.1%) in men and 6.3 (95% CI: 0.4, 12.2) per 100,000 population (increase of 75.0%) in women in 2028.

**Figure 6:**
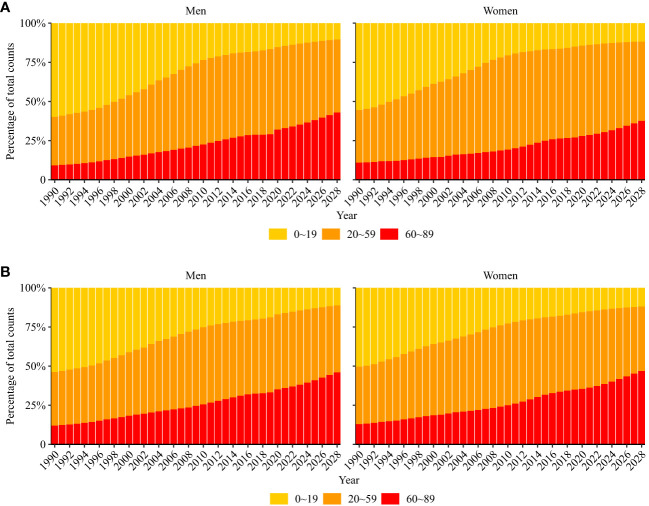
Percentage of modeled and projected acute lymphoid leukemia occurring **(A)** and death **(B)** in three age groups (0–19, 20–59, and 60–89 years).

Mortality due to ALL is projected to decrease from 0.9 (95% CI: 0.9, 1.0) per 100,000 population to 0.8 (95% CI: 0.3, 1.2) per 100,000 population (decrease of 11.1%) in men and 0.7 (95% CI: 0.7,0.7) per 100,000 population to 0.6 (95% CI: 0.2, 0.9) per 100,000 population (decrease of 14.3%) in women from 2019 to 2028. Older adult/adults individuals were projected to have an increasing proportion of incident ALL and ALL-related deaths for both men and women (**Figure 6**).

## 4 Discussion

To our knowledge, this is the first study to assess the long-term temporal trends of ALL incidence and mortality in China, examine the underlying age-, period-, and cohort-specific effects on these trends, and make future predictions to decompose the age composition of the future ALL rate. Our results indicated that ALL incidence and mortality have generally increased in China in the past three decades. Regarding period effects, the incidence and mortality showed upward trends in recent years in both sexes. Regarding cohort effects, the incidence continued to increase as the birth cohort progressed in both sexes (people born later had a higher risk of incident ALL than those born earlier). However, mortality was decreased in the recent birth cohort. It is expected that the incidence rate will continue to increase, but the mortality rate will decrease in both sexes. Older adult/adults individuals were projected to have an increasing proportion of both incident ALL and ALL-related death among both sexes.

The results of this study showed that the incidence rate of ALL generally increased in all the studied age groups in the past 30 years. The increase in the incidence of ALL may be related to the increased exposure to risk factors. Smoking is the most important risk factor leading to ALL-related disability-adjusted life years and death (9). China had one of the highest rates of global tobacco consumption and production, accounting for 40% of global tobacco consumption (17). Although the smoking rate in China has not changed considerably in the last decade (18), tobacco consumption is increasing. The sales volume increased from 76.92 billion packets in 2000 to 127.48 billion in 2014 (19). High body mass index (BMI) is another important risk factor for ALL (9). In the past 40 years, the prevalence of obesity in China has risen rapidly. According to the latest national data from 2015 to 2019, the prevalence of overweight and obesity in Chinese adults reached 34.3% and 16.4%, respectively (20). Occupational exposure may also increase the risk of ALL to some extent. With the rapid development of China’s economy, more people participate in industrial production. In production activities, direct or indirect exposure to hydrocarbons, such as benzene, gasoline, and trichloroethylene, may be related to the occurrence of ALL (21); in addition, parental occupation may also increase the risk of ALL in children (22). Environmental exposure (e.g., benzene (21)) may also contribute to the increase in the incidence of ALL in China. In this study, the mortality due to ALL in children showed a general decreasing trend over the last 30 years, which may be related to the improvements in medical care techniques. Currently, the 5-year overall survival rate in children with ALL is approximately 90% (23).

Age is an important risk factor for ALL. This study indicated that in the same birth cohort, the incidence and mortality of ALL showed a U-shaped correlation with age in both sexes. In children, the immune system is underdeveloped, which leads to their higher susceptibility to bacterial and viral infections than adults when exposed to the same risk factors (24). Some cancer-related somatic mutations occur early in life; for example, approximately 1% of newborns have fusion events of *TEL-AML1* and *AML1-ETO* genes related to leukemia (25). In adults, large chromosome abnormalities (>2 Mbp) associated with leukemia (e.g., aneuploidy and copy-neutral loss of heterozygosity) occur less frequently in those aged<50 years; however, they have a higher prevalence rate in older adult/adults individuals (25), which may contribute to the higher incidence in older adult/adults individuals. Additionally, cumulative exposure to risk factors may also be responsible for the increased incidence and mortality of ALL in older adult/adults individuals. In this study, we found that the incidence and mortality in men were higher than those in women after middle age, which is consistent with previous studies (8). These sex differences may be attributed to the differences in occupations between sexes. In addition, some risk factors for ALL are different between sexes (e.g., smoking and high BMI). In 2003, the prevalence of smoking among Chinese men was 16 times higher than that among women and 17 times higher than that in 2013 (18). Regarding BMI, whether overweight or obese, Chinese men have experienced more significant increases in BMI than women in recent decades (26).

The period effect reflects the different incidence or mortality risk of ALL for different time periods. The results show that period RRs of incident ALL showed monotonic increasing trends in both sexes after 1995, and the period RRs for mortality in both sexes showed upward trends after 2000. This may be related to the above-described increased exposure to ALL risk factors. The cohort effect reflects the different incidence or mortality risk in different birth cohorts. The results showed that the risks of incident ALL in both men and women increased as the birth cohort progressed (people born late had a higher risk of ALL incidence). These results were mainly attributed to increases in ALL risk factors. Considering the impacts of these unfavorable periods and cohort effects, it is expected that the incidence of ALL in China will continue to increase in the future, and it is necessary to control risk factors for ALL. One positive finding was the cohort effect, as mortality due to ALL has continued to decrease in recent-birth cohorts. These favorable trends may be attributed to the above-described improvements in medical care technologies. For the treatment of ALL, new approaches, such as chimeric antigen receptor T cell (CAR-T) therapy, could effectively improve the survival rate in ALL patients. Under these favorable effects, it is expected that mortality due to ALL will decrease in both sexes in China. It should be noted that the proportion of older adult/adults individuals with incident ALL and ALL-related death will gradually increase in the future, which means that more attention should be given to older adult/adults individuals.

Some limitations of the present study should be noted. First, the integrity and accuracy of the ALL data may lead to some bias. Although the GBD 2019 has applied many corrections and adjustments to enhance the comparability of data, including incompleteness, underreporting, and misclassification corrections (27), and the redistribution of “garbage” codes, it is still difficult to completely avoid bias (28). However, bias in this study was reduced to a certain extent compared with the study that used original data without taking these correction and adjustment steps (29). Second, similar to other studies using the APC framework, our study may have ecological fallacies. This limitation is inevitable because the interpretation of the results at the macro level may not be applicable at the individual level. Therefore, the relevant hypotheses proposed in our study need to be further confirmed in individual-based studies. Third, the Bayesian APC model provided higher coverage and better performance in projecting incidence and mortality rates; however, the uncertainty interval was relatively large (15), which made the prediction period relatively short.

## 5 Conclusions

In conclusion, in the past 30 years, the incidence of and mortality due to ALL in both sexes in China have generally increased. It is expected that the incidence of ALL in China in both sexes will continue to increase in the future, but associated mortality will decrease. ALL incidence and mortality is projected to gradually increase in older adult/adults individuals in both sexes in the future. Considering that there is no evidence that the aging process will cease in China, ALL may have a large impact on the health of Chinese older adult/adults individuals. More effective efforts are needed.

## Data availability statement

Publicly available datasets were analyzed in this study. This data can be found here: http://ghdx.healthdata.org/gbd-2019.

## Ethics statement

The GBD study uses identified, aggregated data. Therefore, a waiver of informed consent was reviewed and approved by the University of Washington Institutional Review Board.

## Author contributions

The authors’ responsibilities were as follows: RB, ZS and ZD designed the research. RB and WZ performed the data-analysis. ZD, YS, WZ, SL and JS drafted the original manuscript. RB, QT and ZD critically revised the manuscript; and ZS and ZD provided administrative support for the project and had primary responsibility for the final manuscript. All authors contributed to the article and approved the submitted version.
